# TRPML3 and TRPML1/3 differentially regulate unconventional MIF secretion through TMED10 and secretory autophagy

**DOI:** 10.1080/27694127.2026.2740425

**Published:** 2026-10-08

**Authors:** Jiwoo Park, Jin Kwon, Suzi Choi, Hyun Jin Kim

**Affiliations:** Department of Physiology, Sungkyunkwan University School of Medicine, Suwon, Korea

**Keywords:** MIF, PI(3,4)P_2_, secretory autophagy, TMED10-channeled unconventional protein secretion, TRPML1/3, TRPML3

## Abstract

The intracellular Ca^2+^ channel TRPML3 regulates autophagy by multimerization in a phosphoinositide (PIP)-dependent manner; however, its role in other membrane dynamics remains unclear. Here, we report that TRPML3 regulates type III unconventional protein secretion (UcPS) through two distinct pathways: TMED10-channeled UcPS and secretory autophagy. TRPML3 interacted with TMED10 and macrophage migration inhibitory factor (MIF), a signal sequence-lacking pleiotropic cytokine whose secretion mechanism has remained undefined. We found that cytosolic MIF is transported to membrane-enclosed compartments through TMED10-channeled UcPS. Notably, TRPML3, but not TRPML1 or TRPML1/3, promoted TMED10 oligomerization, thereby enhancing MIF secretion in a Ca^2+^-independent manner. Furthermore, once translocated into vesicular compartments, MIF colocalized with LC3, and its secretion was dependent on secretory autophagy. Mechanistically, we identified PI(3,4)P_2_ as a key regulator of secretory autophagy, with TRPML1/3 acting downstream of PI(3,4)P_2_ to mediate Ca^2+^-dependent MIF secretion. Taken together, our findings demonstrate that MIF is a novel cargo of type III UcPS, operating through TMED10-channeled UcPS and secretory autophagy, which are regulated by TRPML3 in a Ca^2+^-independent manner and by the PI(3,4)P_2_-TRPML1/3 axis in a Ca^2+^-dependent manner, respectively.

## Introduction

TRPML3 is an intracellular Ca^2+^-permeable cation channel involved in multiple cellular processes [1–8]. In particular, we have recently reported that TRPML3 regulates autophagy by multimerization in a phosphoinositide (PIP)-dependent manner: the PI3P-TRPML3 homomer regulates autophagosome formation [9] and the PI4P-TRPML1/3 heteromer mediates autophagosome-lysosome fusion [10]. However, whether TRPML3 also contributes to other membrane trafficking events remains unclear.

The conventional protein secretion pathway involves the translocation of proteins from the endoplasmic reticulum (ER) to the Golgi, followed by delivery to the plasma membrane (PM) for subsequent release into the extracellular space [11,12]. Over the past few years, many cytoplasmic and nuclear proteins lacking an N-terminal signal peptide have been found to exit cells through alternative pathways, collectively referred to as unconventional protein secretion (UcPS), which operate independently of the ER-Golgi route [13,14].

The molecular basis of UcPS has been progressively elucidated, leading to the classification of four distinct pathways in eukaryotic cells. In the type I UcPS pathway, proteins like FGF2 are secreted directly across the PM via lipid pores [15]. ATP-binding cassette (ABC) transporters also promote the secretion of proteins such as HSP70, which is classified as a type II pathway [16]. Type III UcPS pathway utilizes various organelles as intracellular vesicle intermediates, including secretory lysosomes, multivesicular bodies, and secretory autophagosomes [17–20]. Type IV UcPS is a pathway in which PM-resident integral membrane proteins like CFTR are translocated into the ER, then bypass the Golgi apparatus on their way to the PM [21]. More recently, transmembrane p24 trafficking protein 10 (TMED10) has been shown to facilitate the entry of many leaderless cargoes, such as IL-1β, into vesicular compartments for secretion via the type III UcPS pathway [22], a process termed TMED10-channeled UcPS [23]. It has also been suggested that these TMED10-mediated vesicular compartments may function as precursors for secretory autophagosomes, potentially facilitating the subsequent transport of cargoes toward the PM [24].

Macrophage migration inhibitory factor (MIF) is a signal sequence-lacking pleiotropic cytokine associated with various diseases, such as cancer, diabetes, and chronic inflammatory conditions [25]. MIF functions in multiple signaling pathways that regulate cellular proliferation and migration [26]. Although MIF is suggested to be secreted by type II UcPS [27], its precise secretion mechanism remains incompletely defined.

In this study, we identify MIF as a previously unrecognized cargo of type III UcPS, including TMED10-channeled UcPS and secretory autophagy, and further demonstrate that TRPML3 plays a central role in coordinating these processes.

## Results

### TRPML3 interacts with TMED10 and MIF

We previously performed split-ubiquitin membrane yeast two-hybrid screening and identified TMED10 as one of the proteins that interact with TRPML3 [4]. To investigate the relationship between TRPML3 and TMED10, we co-expressed GFP-TRPML3 and mCherry-TMED10, and observed partial colocalization between them (Figure 1(A)). We also conducted co-immunoprecipitation, which confirmed the interaction between TRPML3 and TMED10 (Figure 1(B)). Based on this interaction, we hypothesized that TRPML3 may be involved in TMED10-channeled UcPS. Hence, we searched for other interacting proteins of TRPML3 that are known to be secreted via UcPS, and identified MIF. Because it was difficult to observe colocalization between TRPML3 and MIF upon simple overexpression (Figure 1, upper panel), we treated the cells with digitonin to remove cytosolic MIF and observed that TRPML3 colocalizes well with vesicle-associated MIF (Figure 1, lower panel). We further confirmed the interaction between MIF and the N-terminal of TRPML3 using a GST pull-down assay (Figure 1). These data suggest a functional link between TMED10 and MIF, potentially via TMED10-channeled UcPS, based on their interactions with TRPML3.

**Figure 1.**
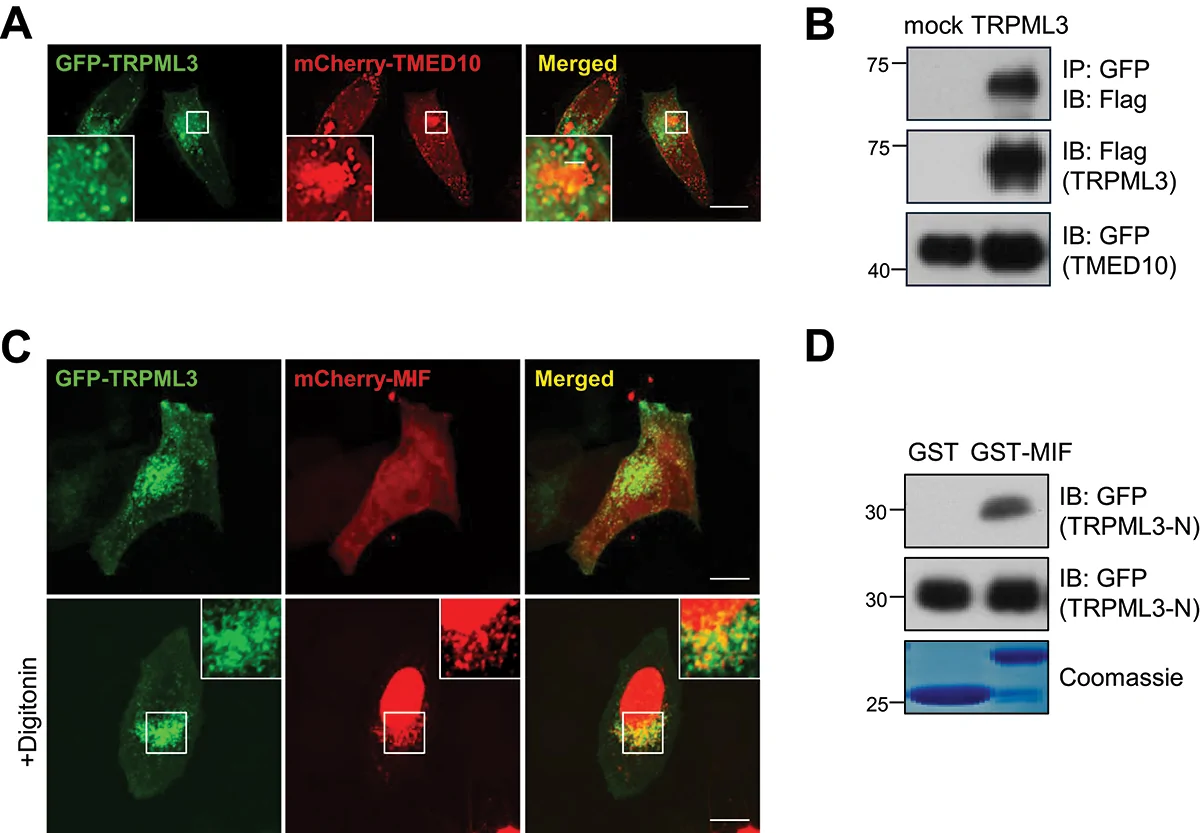
Relationships between TRPML3 and TMED10 or MIF. (A) HeLa cells expressing GFP-TRPML3 and mCherry-TMED10 were fixed and imaged by fluorescence microscopy. Scale bars here and in all other confocal images represent 10 μm. (B) Cell lysates from HEK293T cells expressing mock or Flag-TRPML3 with GFP-TMED10 were subjected to immunoprecipitation using anti-GFP antibody and probed with anti-Flag antibody. (C) HeLa cells co-expressing GFP-TRPML3 and mCherry-MIF were either untreated or treated with cold digitonin (50 µg/ml) and analyzed by confocal microscopy. (D) GST or GST-MIF coupled to glutathione sepharose beads were incubated with whole cell lysates from HEK293T cells expressing GFP-TRPML3-N. Elutes were subjected to SDS-PAGE and immunoblotted with anti-GFP antibody.

### MIF is a substrate of TMED10-channeled UcPS

To confirm the unconventional secretion of MIF, we treated MIF-expressing cells with Brefeldin A, an inhibitor of conventional protein secretion. MIF secretion was not inhibited by Brefeldin A treatment but was even slightly increased, indicating that MIF is not secreted via the conventional protein secretion pathway (Figure 2(A)). Since MIF is known as a cargo of type II UcPS [27], next, we treated the cells with probenecid, a type II UcPS inhibitor. Probenecid inhibited MIF secretion in a dose-dependent manner, but not completely, suggesting that MIF may be secreted through an alternative UcPS pathway (Figure 2(B)). These results, together with those in Figure 1, led us to hypothesize that MIF may be secreted via the TMED10-channeled UcPS. Since TMED10, especially its C-terminal (CT) domain, is known to interact with substrates of TMED10-channeled UcPS [23], we first examined this interaction. A GST pull-down assay revealed that not only full-length but also the CT domain of TMED10 robustly interacted with MIF (Figure 2). This result was also confirmed by the reduced interaction between MIF and TMED10 lacking the CT domain (ΔCT) (Figure 2). In addition, MIF secretion was increased in TMED10-overexpressing cells (Figure 2(E)), whereas it was reduced in TMED10-knockdown cells (Figure 2(F)). Cargoes of TMED10-channeled UcPS regulate TMED10 oligomerization to form a protein channel [23] and their secretion is dependent on RAB1A, RAB1B, and RAB2A [28]. As expected, MIF greatly increased TMED10 oligomerization (Figure 2(G)), and its secretion was increased upon overexpression of RAB1A, RAB1B, and RAB2A (Figure 2(H)). Altogether, these data indicate that MIF is a substrate of TMED10-channeled UcPS.

**Figure 2.**
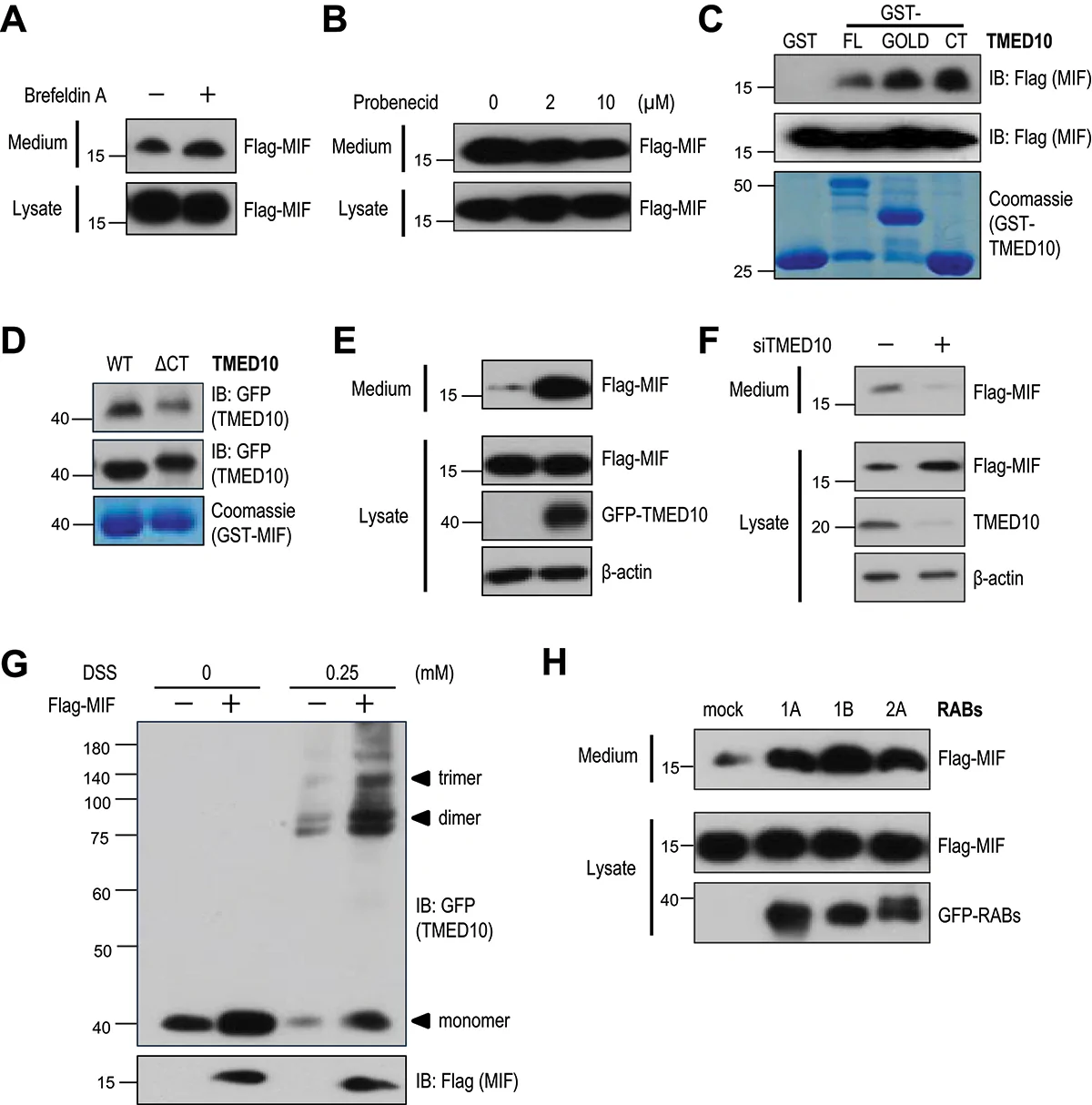
Relationship between MIF and TMED10. (A) Secretion of MIF was measured in HEK293T cells either untreated or treated with brefeldin A. (B) Secretion of MIF was measured in HEK293T cells untreated or treated with the indicated doses of probenecid. (C) GST or the indicated GST-TMED10 domains coupled to glutathione sepharose beads were incubated with whole cell lysates from HEK293T cells expressing Flag-MIF. Elutes were subjected to SDS-PAGE and immunoblotted with an anti-Flag antibody. (D) GST-MIF coupled to glutathione sepharose beads was incubated with whole cell lysate from HEK293T cells expressing GFP-TMED10 or GFP-TMED10(ΔCT). Elutes were subjected to SDS-PAGE and immunoblotted with anti-GFP antibody. (E) Secretion of MIF was measured in HEK293T cells expressing mock or GFP-TMED10. (F) Secretion of MIF was measured in HEK293T cells treated with control siRNA or siRNA targeting TMED10 for 72 h. (G) A crosslink assay was performed in HEK293T cells expressing GFP-TMED10 with mock or Flag-MIF to examine the oligomeric form of TMED10. (H) Secretion of Flag-MIF was measured in HEK293T cells expressing mock, RAB1A, 1B, and 2A.

### TRPML3 regulates TMED10-channeled UcPS in a Ca^2+^-independent manner

In the next step, we sought to clarify the relationship between TRPML3 and TMED10-channeled UcPS. MIF secretion in TRPML3-expressing cells was markedly increased (Figure 3(A), upper panel, lane 2) compared to mock-transfected cells (Figure 3(A), upper panel, lane 1). Surprisingly, however, MIF secretion was not significantly different between WT and dominant-negative (DN) TRPML3 (D458K)-expressing cells (Figure 3(A), upper panel, lane 3). Moreover, TRPML3 activation by 1 μM ML-SA1, a concentration selective for TRPML3 [9], did not substantially affect MIF secretion (Figure 3(A), lower panel), suggesting that TRPML3’s Ca^2+^ channel activity is not required for MIF secretion. Thus, we investigated the effect of the TRPML3 protein itself on TMED10-channeled UcPS, and found that both WT and DN TRPML3 similarly enhanced TMED10 oligomerization even in the absence of cargo (Figure 3(B)). Additionally, no significant difference was observed in the interaction of TMED10 with both WT and DN TRPML3 (Figure 3), indicating that the TMED10-TRPML3 interaction is independent of the channel activity of TRPML3. However, this interaction was completely abolished upon deletion of the CT domain of TMED10, implying that TRPML3 may act similarly to cargoes of TMED10-channeled UcPS (Figure 3). TMED10 oligomerization was observed only when TRPML3 was expressed, but not with TRPML1 or TRPML1/3 (Figure 3(E)), showing the specificity of TRPML3 in regulating TMED10-channeled UcPS. Taken together, these data demonstrate that TRPML3 specifically regulates TMED10-channeled UcPS among TRPML channels by promoting TMED10 oligomerization in a Ca^2+^-independent manner.

**Figure 3.**
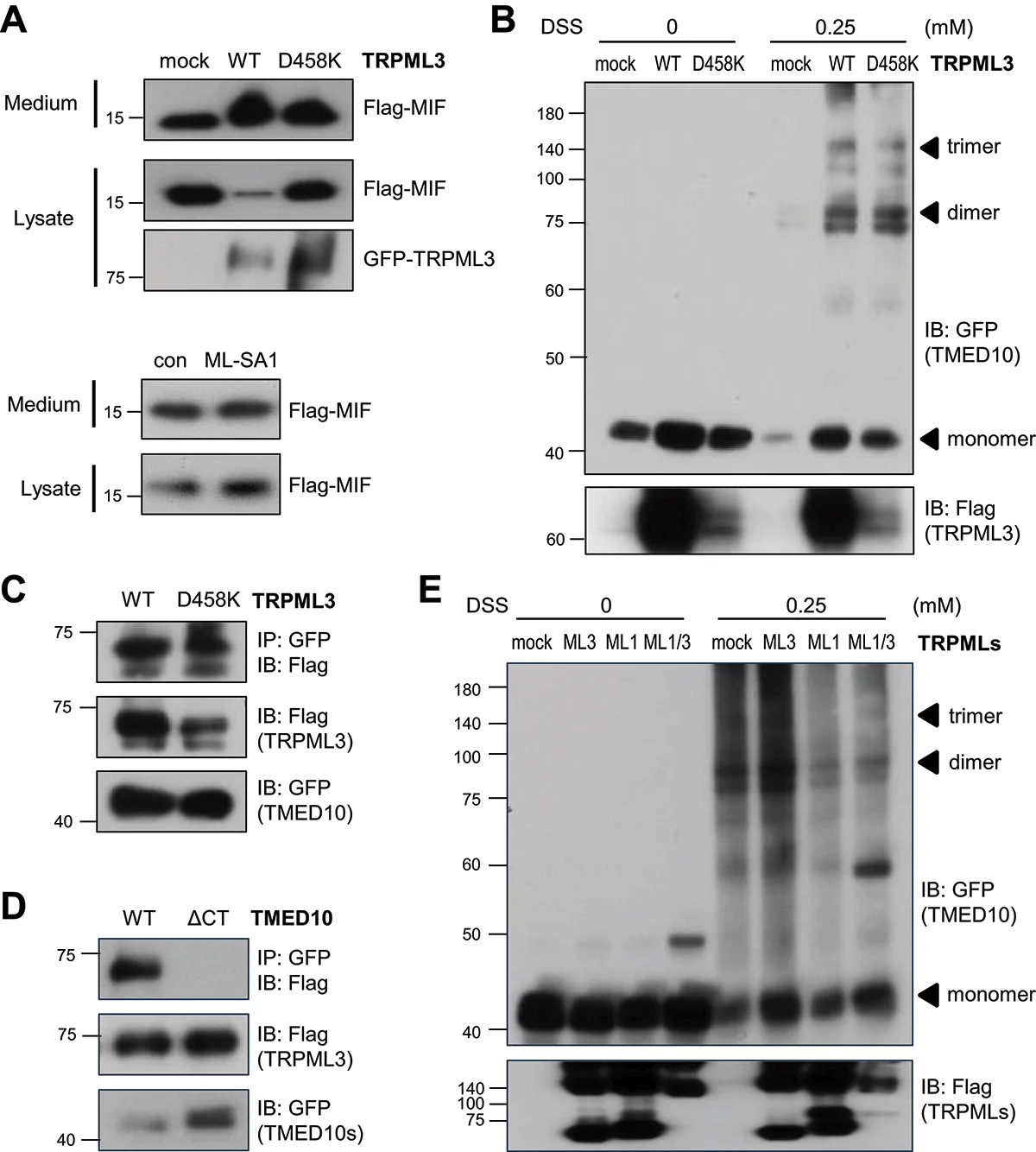
Regulation of TMED10-channeled UcPS by TRPML3. (A, upper) Secretion of MIF was measured in HEK293T cells transfected with mock, GFP-TRPML3, and GFP-TRPML3(D458K). (A, lower) Secretion of MIF was measured in HEK293T cells treated with vehicle or 1 μM ML-SA1. (B) A crosslink assay was performed using HEK293T cells expressing GFP-TMED10 with mock, Flag-TRPML3, and Flag-TRPML3(D458K) to examine the oligomeric form of TMED10. (C) Cell lysates from HEK293T cells expressing wt or D458K Flag-TRPML3 with GFP-TMED10 were subjected to immunoprecipitation with anti-GFP antibody and probed with anti-Flag antibody. (D) Cell lysates from HEK293T cells expressing Flag-TRPML3 with GFP-TMED10 and GFP-TMED10(ΔCT) were subjected to immunoprecipitation with anti-GFP antibody and probed with anti-Flag antibody. (E) a crosslink assay was performed in HEK293T cells expressing GFP-TMED10 with mock, Flag-TRPML3, Flag-TRPML1, and Flag-TRPML1/3 to examine the oligomeric form of TMED10.

### MIF is a cargo of secretory autophagy

Type III UcPS includes not only TMED10-channeled UcPS but also vesicle-mediated pathways such as secretory autophagy [17]. Since we observed colocalization between MIF and TRPML3, which is closely related to autophagy [1,4,6,7,10] (Figure 1(A)), we considered the possibility that MIF could be a cargo of secretory autophagy. Indeed, MIF in vesicular compartments was almost completely colocalized with LC3 (Figure 4(A)). The secretory autophagy pathway also utilizes the same early autophagic machinery as the degradative pathway [29,30]. When early autophagy was inhibited by *ATG5* knockdown or by treating cells with SAR405, a class III PI3K inhibitor, MIF secretion was nearly abolished (Figure 4(B,C)). In contrast, upon inhibition of late autophagy by *SYT5* knockdown [10] or by treating cells with bafilomycin A1, MIF secretion was dramatically increased (Figure 4(D,E)). The increase in MIF secretion is consistent with a previous study showing that MIF secretion is induced by autophagy inhibition [31]. Furthermore, MIF secretion was enhanced by overexpression of SEC22B, a factor specific to secretory autophagy [19], but not to degradative autophagy (Figure 4(F)). We also observed that vesicles containing MIF are closely associated with SEC22B (Figure 4(G)), suggesting that MIF is a cargo of secretory autophagy. To further characterize the identity of MIF-containing vesicles, we examined their localization relative to VAMP8, which has been implicated in lysosome-related and secretory autophagy [32], and VAMP4, a SNARE associated predominantly with TGN-associated trafficking and recycling endosome exocytosis [33,34]. MIF preferentially colocalized with VAMP8 rather than VAMP4 (Figure 4(H)), further supporting the involvement of secretory autophagy-associated vesicular intermediates in MIF trafficking.

**Figure 4.**
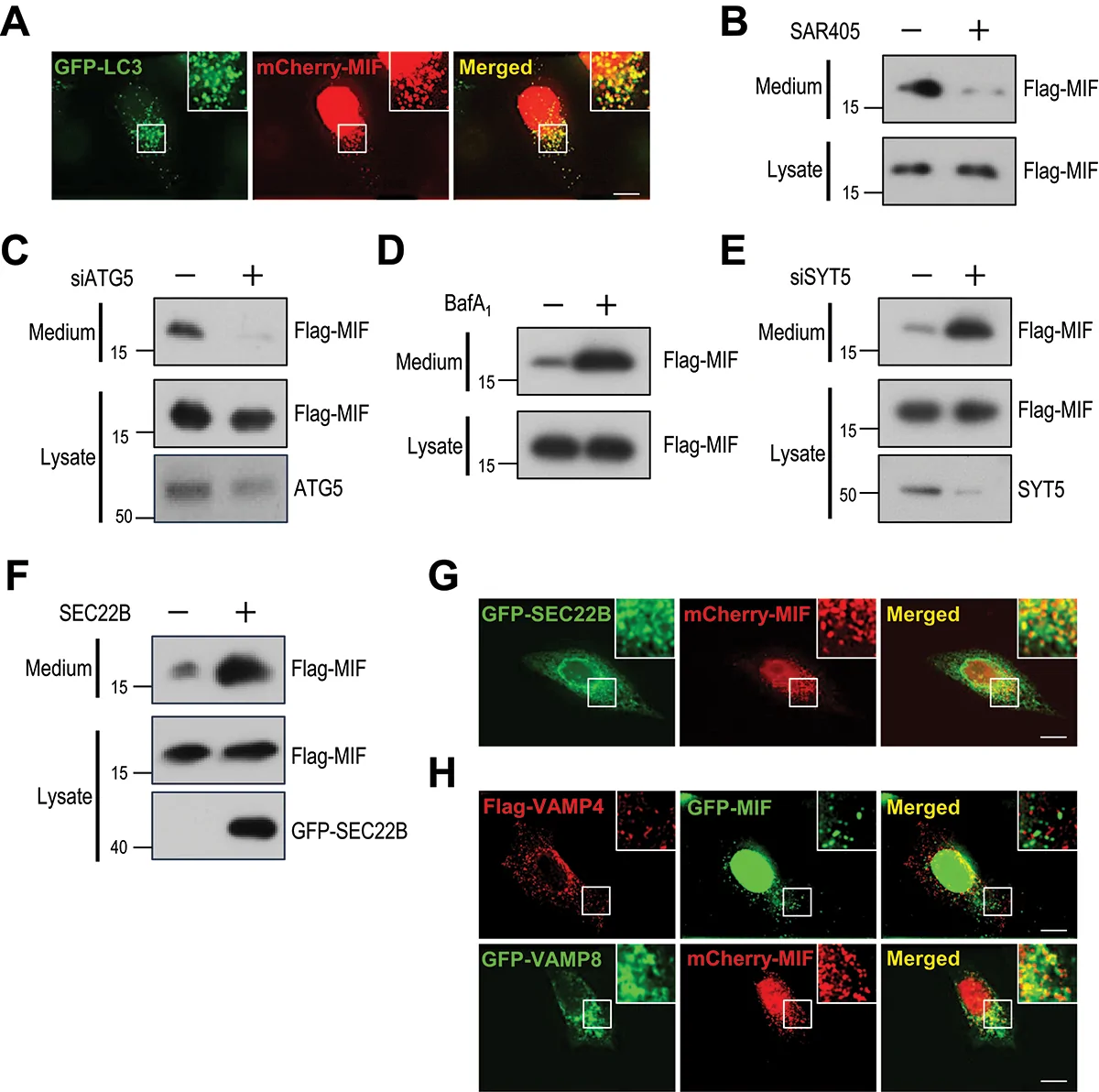
Effect of autophagy on MIF secretion. (A) HeLa cells expressing GFP-LC3 and mCherry-MIF were treated with cold digitonin (50 µg/ml) and imaged by fluorescence microscopy. (B) Secretion of MIF was measured in HEK293T cells either untreated or treated with 10 μM SAR405. (C) Secretion of MIF was measured in HEK293T cells treated with control siRNA or siRNA targeting ATG5 for 72 h. (D) Secretion of MIF was measured in HEK293T cells either untreated or treated with 100 nM bafilomycin A1. (E) Secretion of MIF was measured in HEK293T cells treated with control siRNA or siRNA targeting SYT5 for 48 h. Knockdown efficiency was confirmed by immunoblotting for SYT5. (F) Secretion of Flag-MIF was measured in HEK293T cells expressing mock or GFP-SEC22B. (G) HeLa cells expressing GFP-SEC22B and mCherry-MIF were treated with cold digitonin (50 µg/ml) and imaged by fluorescence microscopy. (H, upper) HeLa cells co-expressing GFP-MIF and Flag-VAMP4 were treated with cold digitonin (50 µg/ml), immunostained with an anti-Flag antibody, and imaged by fluorescence microscopy. (H, lower) HeLa cells expressing GFP-VAMP8 and mCherry-MIF were treated with cold digitonin (50 µg/ml) and imaged by fluorescence microscopy.

### TRPML1/3 functions downstream of PI(3,4)P_2_ in secretory autophagy

Since degradative autophagy is tightly regulated by PIPs, we next aimed to identify the specific PIP species involved in the regulation of secretory autophagy. For this, we used α-synuclein(A53T), a pathogenic mutant form associated with Parkinson’s disease [35], which is a well-known cargo secreted via secretory autophagy [36], but not through the TMED10-channeled UcPS pathway [23]. Secretion of α-synuclein(A53T) was markedly increased by PI(3,4)P_2_ treatment, which was similarly observed for MIF secretion (Figure 5(A,C)), suggesting that PI(3,4)P_2_ regulates secretory autophagy. In degradative autophagy, TRPML channels act as downstream effectors of PIPs: PI3P-TRPML3 and PI4P-TRPML1/3 [9,10]. Thus, we examined which TRPML channels contribute to secretory autophagy and found that TRPML1/3 predominantly increased the secretion of both α-synuclein (A53T) and MIF (Figure 5(B,D)). PI(3,4)P_2_ activated TRPML1/3, as indicated by the increased TRPML1/3-GCaMP6 signal, and concomitantly increased the number of LC3 particles (Figure 5(E,F)), reminiscent of the role of PI4P in degradative autophagy [10]. However, the properties of LC3 particles were distinct, in that PI4P mainly increased acidic autolysosomes linked to degradative autophagy, whereas PI(3,4)P_2_ increased neutral autophagosomes associated with secretory autophagy [30] (Figure 5(G,H)). These results were also confirmed by LC3 western blot analysis (Figure 5).

**Figure 5.**
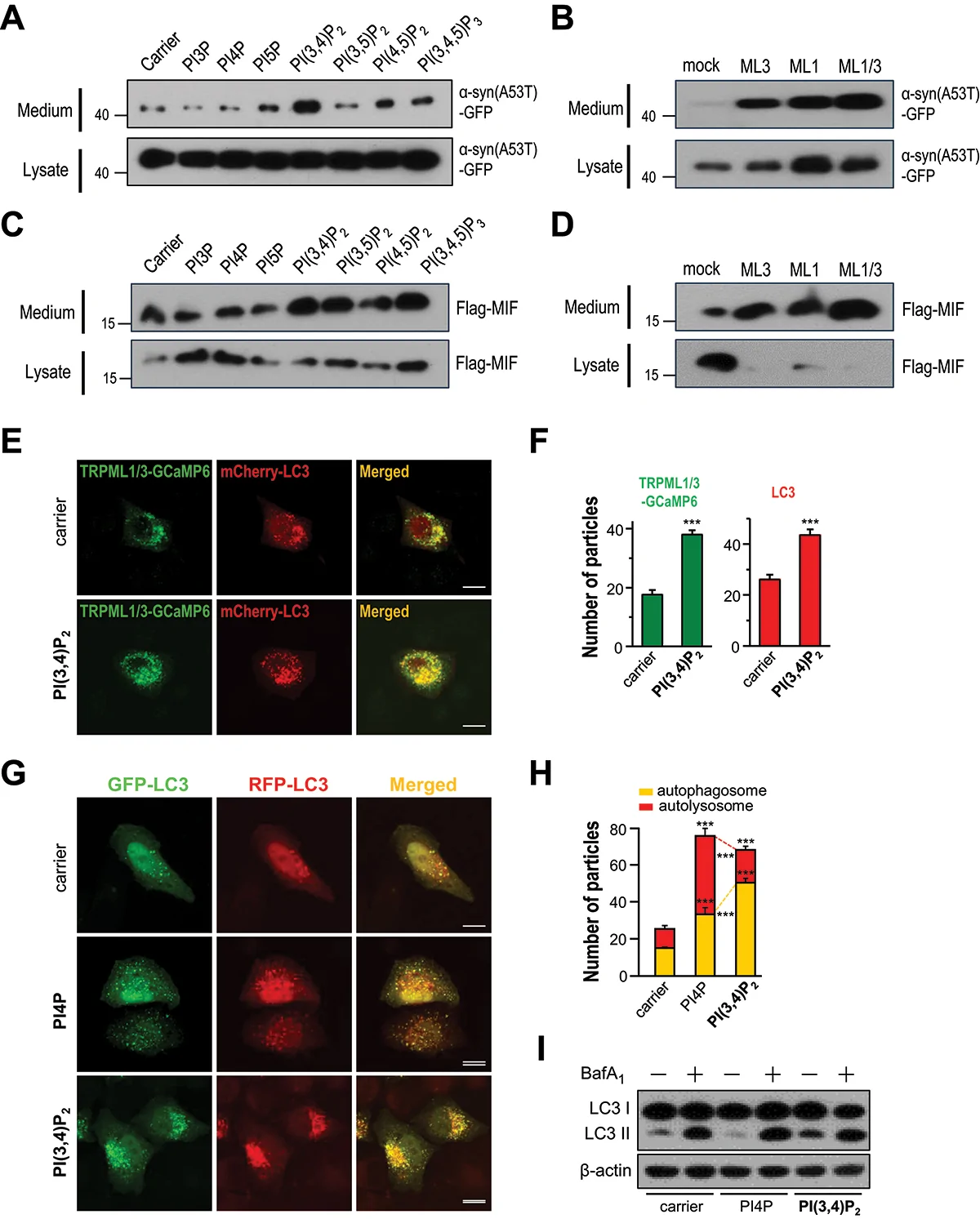
Effect of PIPs and TRPML channels in secretory autophagy. (A) Secretion of α-syn(A53T) was measured in HEK293T cells treated with the indicated 1 μM PIPs for 2 h. (B) Secretion of α-syn(A53T) was measured in Flag-TRPML3, TRPML1, or TRPML1/3 expressing HEK293T cells. (C) Secretion of MIF was measured in HEK293T cells treated with the indicated 1 μM PIPs for 2 h. (D) Secretion of MIF was measured in Flag-TRPML3, TRPML1, or TRPML1/3 overexpressing HEK293T cells. (E) HeLa cells expressing TRPML1/3-GCaMP6 and mCherry-LC3 were treated with either carrier or 1 μM PI(3,4)P_2_ for 1 h and imaged by confocal microscopy. (F) The number of particles of TRPML1/3-GCaMP6 and mCherry-LC3 was counted and is presented as means ± SEM of 19–22 cells (***p < 0.005, Student’s *t*-test). (G) HeLa cells expressing tfLC3 were treated with carrier, 1 μM PI4P, or 1 μM PI(3,4)P_2_ for 1 h and imaged by confocal microscopy. (H) The number of particles of autophagosomes (yellow) and autolysosomes (red) in panel (G) was counted and given as mean ± SEM of 9–12 cells (***p < 0.005, Student’s *t*-test). (I) HEK293T cells were treated with carrier or the indicated PIPs and vehicle or 100 nM bafilomycin A1 for 4 h and processed for Western blot analysis to assay endogenous LC3 levels.

To test whether TRPML1/3 functions downstream of PI(3,4)P_2_, we generated a PIP-binding mutant by substituting eight positively charged residues in TRPML1/3 with glutamine residues (referred to as 8Q), which are critical for PIP–protein interactions [37]. Secretion of α-synuclein(A53T) and MIF was greatly reduced by these charge-removal mutations, and more importantly, this reduction was not rescued even in the presence of excess PI(3,4)P_2_ (Figure 6(A,C)). Finally, secretory autophagy was almost completely blocked by a DN TRPML1/3 mutation (Figure 6(B,D)), indicating that Ca^2+^ release via TRPML1/3 is critical for secretory autophagy. Taken together, these findings suggest that TRPML1/3 acts as a downstream effector of PI(3,4)P_2_ to provide Ca^2+^ required for secretory autophagy.

**Figure 6.**
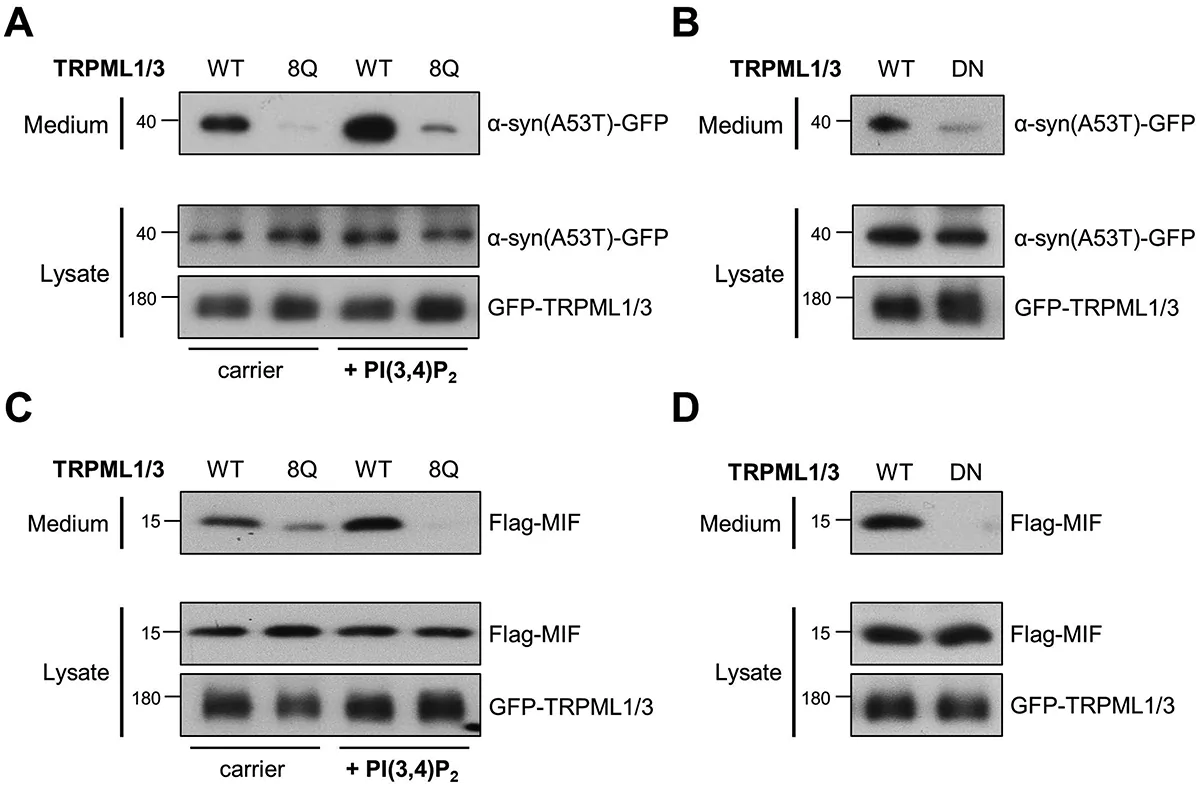
Effect of channel activity and PIP binding in TRPML1/3 on secretory autophagy. (A) Secretion of α-syn(A53T) was measured in HEK293T cells expressing wt or 8Q Flag-TRPML1/3 treated with carrier or 1 μM PI(3,4)P_2_ for 2 h. (B) Secretion of α-syn(A53T) was measured in wt or DN Flag-TRPML1/3 expressing HEK293T cells. (C) Secretion of MIF was measured in HEK293T cells expressing wt or 8Q Flag-TRPML1/3 treated with carrier or 1 μM PI(3,4)P_2_ for 2 h. (D) Secretion of MIF was measured in wt or DN Flag-TRPML1/3 expressing HEK293T cells.

## Discussion

The intracellular Ca^2+^ channel TRPML3 is well-characterized as an essential regulator of autophagy [1,7,38–40]. In this study, we sought to identify a novel function of TRPML3 in other types of membrane dynamics using our previously established TRPML3 interactome (Figure 1) and found that TRPML3 plays a crucial role in TMED10-channeled UcPS (Figure 3). Surprisingly, the Ca^2+^ channel activity was not required for its role in TMED10-channeled UcPS; instead, this process depended on the interaction between TRPML3 and TMED10 (Figure 3). This result is in sharp contrast to previous studies of TRPML3, as it suggests that TRPML3 protein itself possesses a distinct function beyond its role as an ion channel by acting in a cargo-like manner. This finding raises the possibility that TRPML3 may exert additional functions, potentially including those in acidic compartments. Further studies will be needed to determine whether TRPML3 plays such a role in acidic compartments.

In this study, we also identified MIF as a novel cargo of TMED10-channeled UcPS among TRPML3-interacting proteins (Figures 1 and 2). Although MIF does not contain an obvious sequence corresponding to the reported TMED10 recognition motif within mIL-1β [23], motif analysis using the MEME Suite [41] identified a region with sequence similarity to the reported motif (mIL-1β: ^236^NWYIST; MIF: ^108^GWNNST). Whether this putative motif contributes to TMED10-mediated MIF translocation remains to be determined. Given that cargo translocation via TMED10 is considered an early step in secretory autophagy [24], our findings suggest that MIF is subsequently secreted through secretory autophagy (Figure 4), representing the first report of this pathway for MIF. A previous study reported that MIF secretion can also be induced by autophagy inhibition through a mitoROS-dependent mechanism [31]. Consistent with this finding, we observed that inhibition of late autophagy markedly increases MIF secretion (Figure 4(D,E)), suggesting that MIF secretion may be regulated differently depending on the cellular context and the stage of autophagy affected. We reported that TRPML3 forms not only a homotetramer but also a heterotetramer with TRPML1, and these two TRPML3 channels (TRPML3 & TRPML1/3) play distinct roles in degradative autophagy in a PIP-dependent manner [38,42]. Thus, we asked which TRPML3-containing channels and PIPs regulate secretory autophagy, and found that TRPML1/3 functions downstream of PI(3,4)P_2_ in secretory autophagy (Figures 5 and 6).

Importantly, a previous study has shown that TRPML1 does not respond to PI(3,4)P_2_ [43], and our earlier work indicated that TRPML3 is not activated by PI(3,4)P_2_ [9]. In contrast, we recently reported that the TRPML1/3 heteromer can be activated by PI(3,4)P_2_ with PI4P [42]. These observations are consistent with the idea that PI(3,4)P_2_ preferentially engages TRPML1/3, but not TRPML1 or TRPML3, providing a possible mechanistic basis for the PI(3,4)P_2_-TRPML1/3 axis identified in this study. This also raises the question of how the same channel, TRPML1/3, can function downstream of two distinct PIPs, PI4P and PI(3,4)P_2_. We noticed that these two PIPs differentially increase the number of autophagic structures: PI4P predominantly promotes autolysosome formation, whereas PI(3,4)P_2_ increases secretory autophagosomes (Figure 5). Therefore, we conclude that, unlike in degradative autophagy, TRPML1/3 acts at distinct compartments, the secretory autophagosome, as a downstream effector of PI(3,4)P_2_ to promote secretory autophagy. In contrast to TMED10-channeled UcPS, secretory autophagy requires Ca^2+^ release via TRPML1/3, as evident from the result in Figure 6. Since TRPML1/3 is not involved in TMED10-channeled UcPS (Figure 3), the PI(3,4)P_2_-TRPML1/3 axis is specific to secretory autophagy.

Although our data demonstrate that Ca^2+^ release via TRPML1/3 is required for secretory autophagy, the precise downstream processes remain to be defined. One possibility is that TRPML1/3-mediated Ca^2+^ signals facilitate membrane fusion events during secretory autophagy, potentially through the regulation of SNARE proteins such as SEC22B via Ca^2+^ sensor proteins. Further studies will be needed to determine how PI(3,4)P_2_-TRPML1/3-mediated Ca^2+^ signals are deciphered in secretory autophagy.

In conclusion, we identify MIF as a novel cargo of type III UcPS, including TMED10-channeled UcPS and secretory autophagy. In the early stage, MIF translocation into the vesicular compartments via TMED10-channeled UcPS is regulated by the cargo-like function of TRPML3, independent of its Ca^2+^ channel activity. By contrast, in the late stage, MIF secretion via secretory autophagy is controlled by PI(3,4)P_2_-TRPML1/3-mediated Ca^2+^ release. Together, TRPML3 regulates both early and late stages of Type III UcPS through homo- and heteromerization. Given the growing importance of MIF in cancers and inflammatory diseases, the TMED10-TRPML3-MIF axis identified here may represent a potential regulatory pathway linking unconventional MIF secretion to disease progression. Further studies will be needed to determine whether dysregulation of this axis contributes to MIF-driven pathological processes.

## Materials and methods

### Plasmid construction, mutagenesis

pEGFPC1-TRPML3, p3XFlag-CMV-7.1-TRPML3, pEGFPC1-TRPML3(D458K), p3XFlag-CMV-7.1-TRPML3(D458K), pEGFPC1-TRPML1/3, p3XFlag-CMV-7.1-TRPML1/3, pEGFPC1-TRPML1/3(D458K), TRPML1/3-GCaMP6, p3XFlag-CMV-7.1-TRPML1, pGEX-4T1-TRPML3-NΔ30 and pEGFPC1-LC3 were prepared as described previously [1,3,4,6,9,42]. ptfLC3 was a gift from Tamotsu Yoshimori (Addgene 21,074; http://n2t.net/addgene:21074; RRID: Addgene_21074). The coding sequences of SEC22B, TMED10, MIF, VAMP4, and VAMP8 were amplified by PCR and cloned into designated vectors. SEC22B was cloned in-frame into the pEGFPC1 vector using EcoRI and SalI. MIF was cloned in-frame into p3XFLAG-CMV-7.1-mCherry, p3XFLAG-CMV-7.1, and pGEX-4T1 vectors using EcoRI and SalI. TMED10 was cloned in-frame into pEGFPC1, p3XFLAG-CMV-7.1-mCherry, and pGEX-4T1 vectors using EcoRI and SalI. VAMP4 was cloned in-frame into the p3XFLAG-CMV-7.1 vector, and VAMP8 was cloned in-frame into the pEGFP-C2 vector, both using EcoRI and SalI. GST-fusion proteins (GST-TMED10, GST-MIF) were produced using the pGEX-4T1 plasmid (Amersham Biosciences). The coding sequences of GOLD (aa 32–132) and CT (aa 207–219) were cloned in-frame into the pGEX-4T1 plasmid using EcoRI and SalI sites for GST-tagged recombinant protein production (GST-GOLD, GST-CT). To generate GFP-RABs, the coding sequences of RAB1A, RAB1B, and RAB2A were amplified by PCR and cloned into the pEGFPC1 vector using SalI and BamHI for RAB1A, and EcoRI and SalI for RAB1B and RAB2A. The coding sequence of α-synuclein-GFP was amplified by PCR and cloned into the pEGFPN1 vector using BglII and SalI. GFP-TRPML3-N, GFP-TRPML1/3(8Q), and α-synuclein(A53T), along with all other deletion, truncation, and point mutants, were generated by site-directed mutagenesis using nPfu-Forte (Enzynomics, P410). The TRPML1/3(8Q) mutant contains the following substitutions: K59Q, R61Q, R62Q, R65Q in the TRPML1 sequence, and R56Q, R58Q, K59Q, K62Q in the TRPML3 sequence. For knockdown experiments, siRNA targeting human TMED10 (BIONEER, 10,972–1), human ATG5 (BIONEER, 9474–1), human SYT5 (BIONEER, 6861–1), and a negative control scrambled siRNA (Santa Cruz Biotechnology, sc-37007) were used.

### Antibodies and reagents

The following antibodies were used: anti-GFP antibody (Invitrogen, A11122), HRP-Flag antibody (Sigma-Aldrich, A8592), anti-TMED10 antibody (Proteintech, 67,876–1-Ig), anti-ATG5 antibody (Invitrogen, PA1-46178), anti-LC3 antibody (Thermo Fisher Scientific, PA1-16930), and HRP-conjugated β-actin antibody (Sigma-Aldrich, A3854). Other reagents used were Brefeldin A (Toronto Research Chemicals, B677240), Probenecid (Sigma-Aldrich, P8761), SAR405 (Glixx Laboratories, GLXC-05770), bafilomycin A_1_ (Merck Millipore, 196,000) Trichloroacetic acid (Sigma, T6399), DSS (Sigma-Aldrich, S1885), Digitonin (Sigma-Aldrich, D141), protein A agarose beads (Santa Cruz Biotechnology, SC-2001) and glutathione-Sepharose beads (Cytiva, 17,075,601).

### Cell culture and transfection

HEK293T and HeLa cells were maintained in DMEM (Sigma, D5796) supplemented with 10% fetal bovine serum (FBS; Gibco, 16,000,044) and 1% antibiotics (Gibco, 15,240,062) in a humidified incubator at 37°C and 5% CO_2_. For plasmid DNA transfection, cells were plated 1 day before transfection and transfected with Lipofectamine 2000 (Invitrogen, 11,668,019) according to the manufacturer’s instructions. siRNA for *ATG5, TMED10, SYT5*, and negative control scramble siRNA were transfected with Lipofectamine 2000 according to the manufacturer’s instructions.

### Western blotting

Protein samples were separated by SDS-PAGE and transferred to a hydrophobic polyvinylidene difluoride (PVDF) membrane. Membranes were blocked in 5% skim milk in Tween Tris-Buffered Saline (TTBS; pH 7.4, 25 mM Tris, 3 mM KCl, 140 mM NaCl, 0.1% Tween 20) for 1 h at RT and then incubated in specified antibodies for 18 h at 4°C. After three washes, the membranes were incubated for 2 h at RT with HRP-conjugated secondary antibody and HRP-conjugated β-actin antibody (1:10000; Sigma, A3854). Blots were developed using an ECL reagent (GE Healthcare, RPN2232).

### Co-immunoprecipitation

Transfected HEK 293T cells were lysed using 300 µL lysis buffer (50 mM HEPES, 150 mM NaCl, 2 mM MgCl_2_, 1 mM NaF, 1 mM Na_3_VO_4_, 1 tablet of protease inhibitor cocktail, and with 1% Triton X-100 for whole cell lysate). The lysate was incubated on ice for 30 min after sonication and incubated in a rotator at 4°C for 30 min, and any insoluble material was discarded by centrifugation at 14,000 rpm for 20 min. For Co-IP, an anti-GFP antibody was added to each whole cell lysate and incubated for 30 min at 4°C. Then, 25 µL of a 50% slurry of protein A agarose bead was added to the lysate-antibody mixture and further rotated overnight at 4°C. Beads were washed five times with 0.5% Triton-X-100 in PBS and eluted with 5 × sample buffer by heating at 60°C for 5 min. Samples were analyzed by SDS–PAGE, and then transferred to a 0.45 µm PVDF membrane, and blotted with the appropriate antibodies.

### GST pull-down assay

GST-MIF and GST-TMED10s (full-length, GOLD, CT) were expressed in BL21. Extraction of the fusion proteins was initiated by adding 0.4 mM isopropyl β-D-1-thiogalactopyranoside to the bacterial cultures grown to an A600 of 0.6. Bacteria were lysed by sonication for 3 min at an amplitude 40% and three cycles of rapid freezing/thawing. Next, insoluble material was removed by centrifugation for 10 min at 10,000 g. The extract of the fusion proteins was then incubated with glutathione-sepharose beads (GE Healthcare) for 1 h at 4°C. After five washes with 0.5% Triton X-100 in PBS, the protein bound to the beads was eluted in 5 × sample buffer for 5 min at 60°C and analyzed by 10% SDS-PAGE and stained with Coomassie blue. HEK293T cells expressing GFP-tagged proteins and Flag-tagged proteins were lysed 24 h after transfection. The GST-tagged proteins shown above were incubated overnight at 4°C with glutathione-sepharose beads filled with GST-tagged protein. The beads were harvested by centrifugation and washed five times with 0.5% Triton X-100 in PBS. Purified GST fusion proteins were eluted with 5 × sample buffer. Samples were resolved by SDS-PAGE, transferred to PVDF membrane, and blotted with appropriate antibodies.

### Confocal microscopy and immunocytochemistry

HeLa cells transfected with the indicated constructs were grown on glass coverslips and fixed by 4% paraformaldehyde at RT for 20 min. To remove cytosolic protein, cells were permeabilized with 50 µg/ml digitonin in KHM buffer (125 mM potassium acetate, 2.5 mM magnesium acetate, and 25 mM HEPES, pH 7.2) for 3 min at RT and washed twice with KHM buffer before the 4% PFA fixation. After washing, coverslips were mounted on glass slides and analyzed with a Zeiss LSM 710 confocal microscope or Metamorph version 7.8.2.0. The images were analyzed offline using NIH ImageJ^TM^ software (National Institutes of Health).

### PIP delivery experiments

PIP carriers (Echelon Biosciences, P-PC2 or P-PC3) with the indicated PIPs were added at a 1:1 molar ratio (0.5 or 1 μM final concentration) in a test tube. After brief vortexing, the complex was incubated for 5 min at RT, added to the medium of adherent cells, and incubated for 1 to 2 h at 37°C.

### TCA precipitation

For protein secretion analysis, secreted proteins in the culture medium were concentrated using trichloroacetic acid (TCA) precipitation. The culture medium was replaced with HBSS for 1 hour before collection, and the medium was subsequently clarified by centrifugation at 2,000 × g for 5 min at 4°C. 100% (w/v) TCA was added to the resulting supernatant at one-fourth of the medium volume. After gentle mixing by inversion, the mixture was incubated on ice for 20 min. The precipitated proteins were then pelleted by centrifugation at 17,000 × *g* for 5 min at 4°C. The supernatant was carefully discarded, and the pellet was washed twice with 200 µL of ice-cold acetone. The pellet was air-dried for 5–10 min to remove residual acetone. Finally, the dried pellet was resuspended in 10 µL of lysis buffer and 2.5 µL of 5× sample buffer, and the samples were analyzed by immunoblotting.

### Crosslink assay

For the DSS crosslink assay, transfected HEK293T cells were harvested and resuspended in 600 µL of 1×PBS. Each sample was split in half, and the indicated concentration of DSS dissolved in dimethylsulfoxide was added to half the cells, while the same volume of dimethylsulfoxide was added to the other half as a control. After the incubation for 30 min at room temperature, the crosslinking reaction was quenched with 20 mM Tris for 15 min. Crosslinked cells were lysed with 1% Triton X-100 containing lysis buffer and centrifuged at 15,000 × *g* for 20 min, followed by immunoblot analysis.

#### Statistical analysis

Results are presented as the mean ± standard error of the mean (SEM). Data were analyzed using Origin version 7.0. Normality was assessed using the Shapiro–Wilk test. Statistical comparisons between two groups were performed using Student’s *t*-test. Differences were considered statistically significant at *p* < 0.05.

## Data Availability

All data related to the study is presented in supplementary material.
